# FasL microgels induce immune acceptance of islet allografts in nonhuman primates

**DOI:** 10.1126/sciadv.abm9881

**Published:** 2022-05-13

**Authors:** Ji Lei, María M. Coronel, Esma S. Yolcu, Hongping Deng, Orlando Grimany-Nuno, Michael D. Hunckler, Vahap Ulker, Zhihong Yang, Kang M. Lee, Alexander Zhang, Hao Luo, Cole W. Peters, Zhongliang Zou, Tao Chen, Zhenjuan Wang, Colleen S. McCoy, Ivy A. Rosales, James F. Markmann, Haval Shirwan, Andrés J. García

**Affiliations:** 1 Center for Transplantation Science, Massachusetts General Hospital, Harvard Medical School, Boston, MA, USA.; 2 Woodruff School of Mechanical Engineering and Petit Institute for Bioengineering and Bioscience, Georgia Institute of Technology, Atlanta, GA, USA.; 3 Departments of Child Health and Molecular Microbiology and Immunology, School of Medicine, University of Missouri, Columbia, MO, USA.; 4 Department of Microbiology and Immunology, Institute for Cellular Therapeutics, University of Louisville, Louisville, KY, USA.; 5 Department of General Surgery, General Hospital of Western Theater Command, Chengdu, China.; 6 Cellular Therapy Department, Xiang’an Hospital, Xiamen University Medical School, Xiamen, China.; 7 Division of Comparative Medicine, Massachusetts Institute of Technology, Boston, MA, USA.

## Abstract

Islet transplantation to treat insulin-dependent diabetes is greatly limited by the need for maintenance immunosuppression. We report a strategy through which cotransplantation of allogeneic islets and streptavidin (SA)–FasL–presenting microgels to the omentum under transient rapamycin monotherapy resulted in robust glycemic control, sustained C-peptide levels, and graft survival in diabetic nonhuman primates for >6 months. Surgical extraction of the graft resulted in prompt hyperglycemia. In contrast, animals receiving microgels without SA-FasL under the same rapamycin regimen rejected islet grafts acutely. Graft survival was associated with increased number of FoxP3^+^ cells in the graft site with no significant changes in T cell systemic frequencies or responses to donor and third-party antigens, indicating localized tolerance. Recipients of SA-FasL microgels exhibited normal liver and kidney metabolic function, demonstrating safety. This localized immunomodulatory strategy succeeded with unmodified islets and does not require long-term immunosuppression, showing translational potential in β cell replacement for treating type 1 diabetes.

## Teaser

Transplantation of allo-islets and FasL microgels results in glycemic control in diabetic primates without immunosuppression.

## INTRODUCTION

Type 1 diabetes (T1D) is caused by the autoimmune destruction of pancreatic islet β cells (*1*). Its prevalence in the United States has risen steadily since the 1980s, with T1D afflicting >1.5 million Americans (*2*). Transplantation of pancreatic islets from cadaveric donors offers restoration of β cell mass in T1D patients with the potential for prevention and reversal of diabetic complications (*3*). Nonetheless, islet graft recipients must be immunosuppressed for the rest of their lives with agents that not only are toxic to the recipient and graft β cells but also may induce peripheral insulin resistance (*4*). Thus, the development of tolerogenic regimens that obviate the need for immunosuppression will facilitate the broad application of islet transplantation as a cure for T1D.

Immune tolerance was first demonstrated in small-animal models more than 6 decades ago (*5*). Intensive efforts assessing various tolerance-inducing strategies (*6*–*8*), such as lymphocyte depletion, costimulatory blockade, and administration of T regulatory cells (T_regs_), tolerogenic dendritic cells, apoptotic donor leukocytes, and myeloid-derived suppressor cells, have not translated into a protocol with efficacy in the clinic. Mixed hematopoietic chimerism has so far been the only protocol to achieve clinical tolerance, but only in a small number of patients (*9*). Whereas donor chimerism–induced transplant tolerance in kidney recipients represents a groundbreaking advance for the field, this approach suffers from the requirement for intensive conditioning to achieve donor chimerism and the potential for acute or chronic graft-versus-host disease (*10*). Similarly, in nonhuman primates (NHPs), the gold standard translational model for allogeneic transplantation, only mixed chimerism has induced tolerance to same-donor kidney allografts (*11*). A recent nonchimeric approach using peritransplant administration of apoptotic donor lymphocytes under the cover of an induction regimen consisting of rapamycin, anti-CD40 and interleukin-6R (IL-6R) antibodies, and soluble tumor necrosis factor receptor (TNFR) reported ~1-year islet allograft survival in NHPs (*8*). However, the clinical application of this protocol may be limited as graft survival was achieved only in single major histocompatibility complex (MHC) class II *DRB* allele-matched recipients and required one dose of cell infusion 1 week before transplantation.

The Fas receptor/Fas ligand (FasL) pathway plays crucial roles in activation-induced cell death and tolerance to self-antigens (*12*). The efficacy of FasL gene therapy to mitigate allogeneic immune responses for graft acceptance has been demonstrated in experimental animal models (*13*, *14*). Nevertheless, the uncertain safety profile of sustained ectopic expression of FasL in target tissues, opposing properties of soluble versus membrane-bound forms of FasL (*15*, *16*), and technical and regulatory challenges of gene therapy severely curtail clinical applicability. To investigate the translational potential of FasL as an immunomodulatory agent, our group constructed a chimeric protein consisting of the extracellular domain of FasL with the core streptavidin protein (SA), SA-FasL. SA-FasL exists as multimers with robust apoptotic activity on Fas-expressing cells and lacks the matrix metalloproteinase–cleavable sites present in its native extracellular domain (*17*). Pancreatic islets chemically modified to present SA-FasL protein on their surface survive indefinitely under the kidney capsule in the absence of chronic immunosuppression in an allogeneic transplant murine model (*18*). However, the requirement for chemical modification of the islet surface to present SA-FasL limits translation of this approach into the clinic. For this reason, we recently engineered poly(ethylene glycol) (PEG)–based synthetic hydrogel microspheres (microgels) for controlled presentation of bioactive SA-FasL (*19*). Cotransplantation of SA-FasL–presenting microgels with unmodified allogeneic islets under transient cover of rapamycin resulted in long-term engraftment and function in diabetic mice. Recipients generated normal systemic responses to donor antigens, implying immune privilege of the graft, and FoxP3^+^ T_regs_ were instrumental in graft acceptance as their deletion resulted in prompt graft rejection. Thus, this biomaterial-based strategy offers off-the-shelf immunomodulatory capability without modification of donor islets, increasing its clinical relevance and breadth of potential clinical applicability. Here, we investigated the immunomodulatory potential of the SA-FasL microgel technology in a preclinical streptozotocin (STZ)–induced diabetes NHP model in which allogeneic islets and SA-FasL microgels are cotransplanted to the omental pouch.

## RESULTS

### SA-FasL microgels promote sustained islet allograft survival and function

**Fig. 1.**
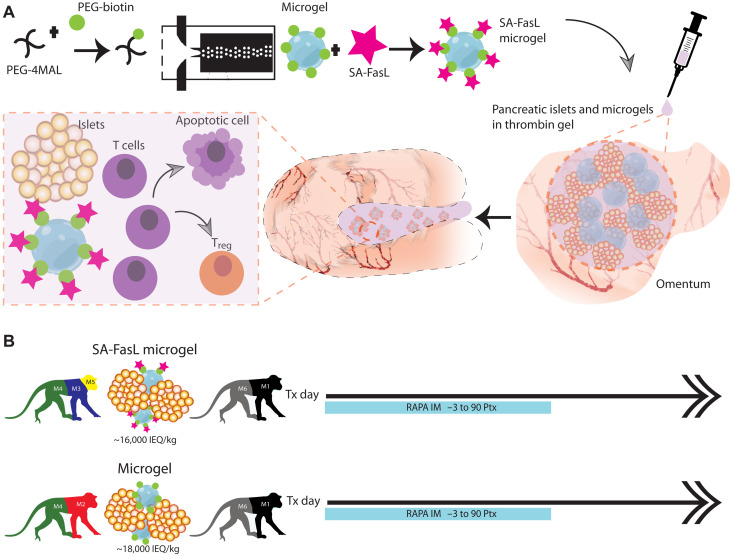
Local presentation of SA-FasL via synthetic microgels for islet transplantation. (**A**) Synthetic biotin-PEG microgels, generated by microfluidic polymerization, capture, and present SA-FasL. SA-FasL–presenting microgels and islets are immobilized on the surface of the omentum of NHPs using autologous plasma and thrombin, where they induce immune acceptance by potentially eliminating T effector cells and generating T regulatory cells. (**B**) Schema describing donor and diabetic MHC mismatched recipient NHPs and treatment protocol. SA-FasL–presenting microgels with transient rapamycin (RAPA) monotherapy (SA-FasL Microgels, *n* = 4); control microgels with transient rapamycin monotherapy (Microgels, *n* = 3).

In the clinic, intraportal administration to lodge islets inside the liver has been used almost exclusively and is the only site with demonstrated efficacy for sustained graft function (*20*). Infusion of islets into the portal system, however, triggers activation of platelets, coagulation factors, and the complement system, resulting in significant acute loss of islets (*21*). The omentum is an alternative site ideal for the colocation of islets and immunomodulatory microgels, providing a capacious site to host the material, rich access to capillary beds, and delivery of insulin into the portal circulation (*22*, *23*). Here, we evaluated SA-FasL–presenting microgels for sustained survival and function of transplanted allogeneic islets transplanted to the omentum with a thrombin-induced fibrin matrix per the Miami protocol (*23*) (Fig. 1A).

We previously demonstrated that cotransplantation of SA-FasL–presenting microgels with allogeneic islets under a transient cover of rapamycin resulted in indefinite graft survival in mice (*19*). This protocol was adopted for the present NHP study except for the rapamycin regimen (as detailed below) because of differences in metabolic and pharmacological dynamics between NHPs and rodents (*24*). For the present study, the rapamycin transient regimen was informed by a combination of toxicity knowledge derived from previous clinical reports (*25*) and our decade-long experience as to what would be tolerable and functional levels in NHPs to achieve possible graft protection. Biotinylated PEG microgels (150 μm diameter) were synthesized by microfluidic polymerization and functionalized with SA-FasL as described (*19*). This synthetic biomaterial provides a highly reproducible platform for controlled presentation of SA-FasL (fig. S1). Four diabetic NHPs (Fig. 1B and tables S1 and S2) underwent transplantation of allogeneic islets [14,800 to 18,700 islet equivalents (IEQ)/kg] mixed with SA-FasL–presenting microgels (150,000 to 200,000 microgels delivering 0.2 mg of SA-FasL) at the omental surface. A 3-month course of rapamycin monotherapy was used—target rapamycin blood trough level of 40 ng/ml for the first 2 weeks and then 20 ng/ml until the 3-month time point after transplantation, when rapamycin was discontinued without weaning. Rapamycin levels became undetectable 2 weeks after discontinuation. We note that our prior studies (*7*, *26*) and other studies (*27*) showed that rapamycin monotherapy could not prevent islet graft rejection even at levels >50 ng/ml in allogeneic NHP models. Three diabetic NHPs (Fig. 1B and tables S1 and S2) had allogeneic islets (19,300 to 20,000 IEQ/kg) cotransplanted with unmodified PEG microgels lacking SA-FasL protein into the omentum under a similar course of rapamycin which served as a control.

**Fig. 2.**
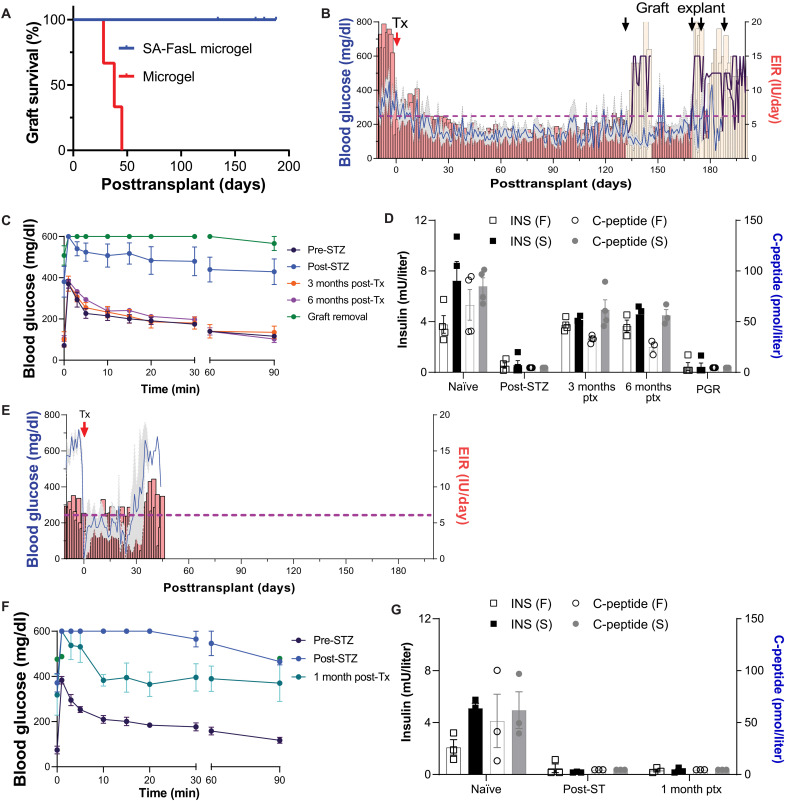
SA-FasL–presenting microgels induce islet allograft acceptance. (**A**) Kaplan-Meier survival curves of islet allografts in SA-FasL Microgel (*n* = 4) and Microgel (*n* = 3); Mantel-Cox test, *P* = 0.010. Mean graft survival times: SA-FasL Microgel, >180 days; Microgel, 27.7 days. (**B**) Nonfasting blood glucose levels (mean, blue line; SEM, gray shadow, left axis) and daily total EIR (mean, red bars; lower SEM, dark red bars, right axis) for SA-FasL Microgel subjects. Animals exhibited high blood glucose levels and external insulin demand after STZ induction but before transplant (defined as post-STZ). After cotransplantation of islets and SA-FasL microgels (Tx), animals rapidly became normoglycemic and had significantly reduced EIR. Animals reverted to hyperglycemic state after graft removal (blood glucose levels, purple lines, left axis; total EIR, tan bars, right axis). (**C**) Blood glucose levels for SA-FasL Microgel subjects after intravenous infusion of glucose before (pre-STZ) and after diabetes induction (post-STZ), at 3 and 6 months after transplantation, and after graft removal. (**D**) Insulin (left axis) and C-peptide (right axis) levels in serum for SA-FasL Microgel animals under fasting (F) and post-stimulation (S) before (naïve) and after diabetes induction, at 3 and 6 months after transplantation, and post-graft removal (PGR). (**E**) Nonfasting blood glucose levels (blue line; SEM gray shadow, left axis) and daily total EIR (mean, red bars; lower SEM, dark red bars, right axis) for Microgel subjects. After restoring normoglycemia following transplantation (Tx), animals became hyperglycemic and required higher external insulin around 1 month after transplantation. (**F**) Blood glucose after intravenous infusion in Microgel during prediabetic state (Pre-STZ), after diabetes induction (Post-STZ), and at 1 month after transplantation. (**G**) Insulin (left axis) and C-peptide (right axis) levels in serum for Microgel animals under fasting (F) and post-stimulation (S) before and after diabetes induction and at 1 month after transplantation.

After islet transplantation, prompt glycemic control was achieved and maintained by all animals receiving SA-FasL–presenting microgels for observation periods >134, >170, >177, and >188 days (Fig. 2, A and B, and figs. S2 and S3). Animals were terminated with functioning grafts due to coronavirus disease 2019 (COVID-19) pandemic–associated institution-imposed logistical barriers. In contrast, control subjects, which received an identical rapamycin regimen and had comparable rapamycin plasma levels (*P* = 0.63; fig. S4, D and E) but no SA-FasL, maintained glycemic control for only 21, 27, and 35 days with a mean survival time of 27.7 days (Fig. 2, A and E, *P* = 0.010, and figs. S3 and S5).

Figure 2B presents nonfasting blood glucose levels (left axis) and administered external total daily insulin requirement (EIR; right axis) averaged across all SA-FasL–presenting microgel recipients; fig. S2 (A to D) depicts data for individual recipients. Before islet transplantation, animals in the SA-FasL Microgel group had average nonfasting blood glucose levels around 400 mg/dl and required 16 to 22 units of exogenous insulin per day. After transplantation, the fasting blood glucose levels were in the normal range for all animals [using intravenous glucose tolerance tests (IVGTT) time 0 reading at 1, 3 and 6 months as subjects were fasted overnight (Fig. 2C)], but postprandial glucose levels for some animals fluctuated between normal and occasionally >300 mg/dl. Nevertheless, SA-FasL Microgel subjects maintained excellent glycemic control within the normal range for the duration of the study (post-STZ/pretransplant versus posttransplant, *P* = 0.0035) (Fig. 2B). Three SA-FasL Microgel subjects required exogenous insulin after transplantation, but only 10 to 20% of the pretransplant dose to maintain postprandial blood glucose levels below 250 mg/dl (fig. S2, A to C). Subject 4 (M9118) initially required one to two units of insulin per day but developed a fully functioning graft requiring occasional exogenous insulin at 4 months after transplantation (fig. S2D). For SA-FasL Microgel subjects, posttransplant EIR was significantly lower than post-STZ/pretransplant (*P* = 0.0092). All animals experienced initial weight loss after STZ induction and after transplantation, but they gradually recovered, indicating that posttransplant euglycemia was not due to malnutrition (fig. S2, E to H). It is expected that both STZ induction and the transplant surgery will cause immediate weight loss. The continued initial minor weight loss after transplantation is most likely associated with rapamycin therapy. Results for metabolic markers indicated normal liver and kidney function for SA-FasL Microgel NHPs (fig. S4, A to C, blue lines). After surgical removal of islet grafts at the end of the study, SA-FasL Microgel animals promptly returned to a diabetic state (blood glucose levels: posttransplant versus post-graft explant, *P* < 0.0001; EIR: posttransplant versus post-graft explant, *P* = 0.0042) (Fig. 2B and fig. S2, A to D), demonstrating that blood glucose control was due to the graft.

At selected time points, IVGTTs were performed to assess glucose disposal kinetics and insulin and C-peptide levels. After glucose bolus challenge, SA-FasL Microgel subjects returned to normal blood glucose levels within 90 min, with comparable profiles to that of naïve animals before diabetes induction [area under the curve (AUC) analysis: pre-STZ (naïve) versus post-STZ (after STZ induction but before transplant), *P* < 0.0001; 3 months after transplant versus post-STZ, *P* < 0.0001; 6 months after transplant versus post-STZ, *P* = 0.0002] (Fig. 2C). After removal of the graft, blood glucose levels remained elevated and equivalent to pretransplant diabetic levels (AUC: post-graft removal versus post-STZ, *P* = 0.0760), demonstrating that the control of blood glucose levels was due to the graft. Consistent with these metrics of graft function, background levels of insulin and C-peptide were measured in the serum of SA-FasL Microgel NHPs after STZ administration but before islet transplant and equivalently low levels were evident after graft removal (insulin: pre-STZ versus post-STZ, *P* < 0.0001; post-STZ versus post-graft removal, *P* = 0.9998; C-peptide: pre-STZ versus post-STZ, *P* = 0.0060; post-STZ versus post-graft removal, *P* = 0.4032) (Fig. 2D). SA-FasL Microgel subjects regained insulin and C-peptide expression (insulin: 3 months after transplant versus post-STZ, *P* = 0.0022; 6 months after transplant versus post-STZ, *P* = 0.0007; C-peptide: 3 months after transplant versus post-STZ, *P* = 0.0248; 6 months after transplant versus post-STZ, *P* = 0.0230), with levels similar to the prediabetic state. Notably, stimulated insulin and C-peptide levels were higher than corresponding fasting levels, indicating glucose responsiveness (insulin: *P* = 0.0274, C-peptide: *P* = 0.0149). Glycated hemoglobin (HbA1c) levels for SA-FasL Microgel subjects were well controlled after transplantation (fig. S2M), with most (three of four) animals with A1C levels below 6% at the end of the study. We detected the presence of SA-FasL protein in the serum of all subjects as early as 1 day after transplantation ranging from 4 to 56 ng/ml and decreasing to background levels by days 7 to 14 (fig. S2, I to L). This result is consistent with prior in vivo measurements of SA-FasL microgels in mice, showing a local half-life of 3.0 days (*19*).

Figure 2E presents random nonfasting blood glucose levels (left axis) and total daily EIR (right axis) averaged across all recipients receiving control microgels (referred to here as simply “Microgel”); data for individual recipients are provided in fig. S5 (A to C). Before islet transplantation, Microgel subjects had average nonfasting blood glucose levels around 400 mg/dl while requiring 15 to 20 units of exogenous insulin per day. After transplantation, Microgel subjects experienced uneventful recovery, and average blood glucose levels returned to the normal range. However, around day 30 after transplantation, blood glucose levels and required external insulin dose increased and remained elevated at levels comparable to the diabetic pretransplantation state, indicating graft rejection (blood glucose levels: posttransplant versus postrejection, *P* = 0.0050; EIR: posttransplant versus post-graft rejection, *P* = 0.0441). After transplantation, animals initially lost weight but then started gaining weight around day 25 (fig. S5, D to F). IVGTT showed poor graft function as glucose levels remained elevated after glucose bolus injection (AUC: pre-STZ versus post-STZ, *P* = 0.0013; 1 month after transplant versus post-STZ, *P* = 0.1036) (Fig. 2F). Similarly, insulin and C-peptide levels at 1 month after transplantation remained at pretransplant diabetic levels (insulin: pre-STZ versus post-STZ, *P* < 0.0001; 1 month after transplant versus post-STZ, *P* = 0.9504; C-peptide: pre-STZ versus post-STZ, *P* < 0.0134; 1 month after transplant versus post-STZ, *P* > 0.9999) (Fig. 2G).

**Fig. 3.**
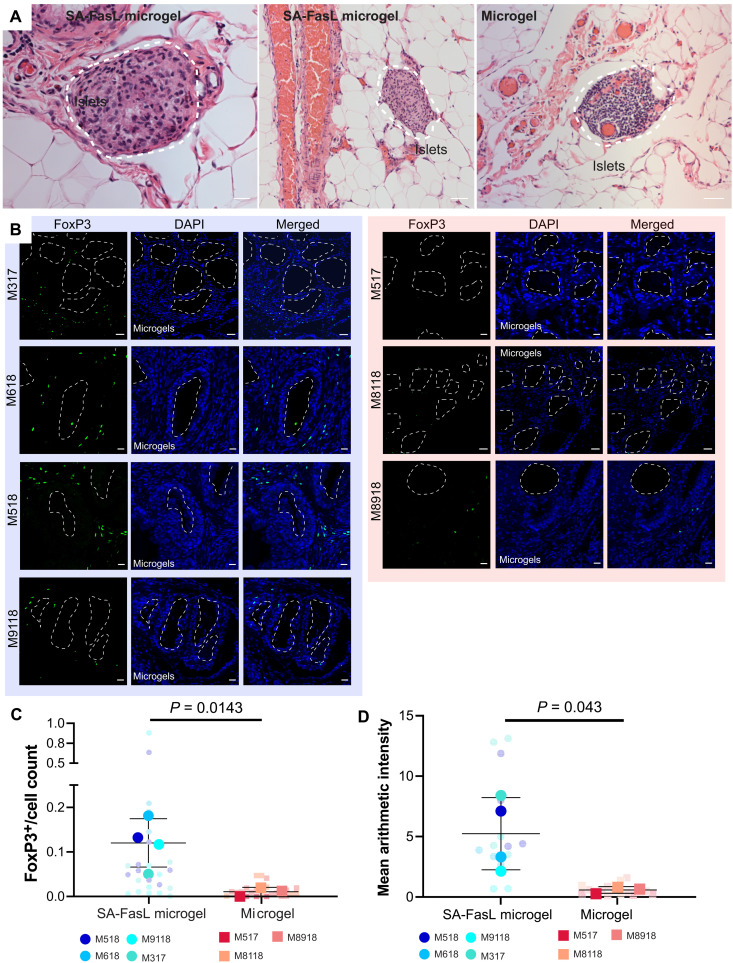
Histological analyses of grafts showing increased FoxP3^+^ cells in recipients of SA-FasL–presenting microgels. (**A**) Hematoxylin and eosin (H&E) staining of excised omentum biopsies at takedown demonstrates the presence of islet-like clusters (outlined in white dashed line) in SA-FasL Microgel subject with minimal immune cell infiltration (left image: bar, 20 μm; middle image: bar, 50 μm). Representative image from Microgel (right image: bar, 50 μm) shows islet-like cluster with significant cell infiltration. (**B**) Immunostaining for FoxP3 [green: FoxP3; blue: 4′,6-diamidino-2-phenylindole (DAPI); bar: 50 μm], a marker of T_regs_, showing increased numbers of FoxP3^+^ at the graft site of animals receiving SA-FasL microgels compared to those receiving microgels. FoxP3^+^ cells were in closed apposition to microgels (dashed white lines). (**C** and **D**) Quantification of FoxP3^+^ cells over total cell counts and mean intensity in sections from Microgel and SA-FasL Microgel animals demonstrating increased frequency of T_regs_ in SA-FasL Microgel subjects (nested two-tailed *t* test). Plots show data points for each animal in a different color, with a minimum of three representative images analyzed per animal.

Autopsy pathology showed normal hematoxylin and eosin (H&E) staining for liver, heart, lung, kidney, and intestinal tissue for SA-FasL Microgel NHPs (fig. S6A). Recipient pancreases contained few small islet-like clusters of endocrine cells but were devoid of insulin-positive β cells (fig. S6B), indicating effective elimination of host β cells by STZ treatment and confirming that posttransplant glycemic control is attributable to the graft. Histological analyses of the omentum transplant site for SA-FasL Microgel NHPs revealed numerous well-granulated clusters of cells reminiscent of islets with minimal to no infiltrating lymphocytes, suggesting the absence of rejection (Fig. 3A). Histology of omentum for Microgel subjects revealed islet-like clusters that were infiltrated by lymphocytes, consistent with graft rejection (Fig. 3A). Immunostaining of the graft site demonstrated well-preserved insulin^+^ structures corresponding to transplanted islets in long-term (day 177) SA-FasL Microgel NHPs, whereas sections for Microgel control NHPs at rejection (day 21) show structures with loss of insulin staining at the periphery (fig. S7). Immunostaining analyses of the graft site demonstrated the presence of FoxP3^+^ (a marker of T_regs_) cells at a higher frequency (cell count, intensity) in SA-FasL Microgel NHPs compared to Microgel subjects (FoxP3^+^ cell counts: *P* = 0.0143; FoxP3^+^ intensity: *P* = 0.043; Fig. 3, B to D). This finding is in agreement with studies in diabetic mice demonstrating a pivotal role for T_regs_ in establishing SA-FasL microgel–induced immune acceptance (*19*).

### SA-FasL microgel recipients display no changes in systemic immune cell populations

**Fig. 4.**
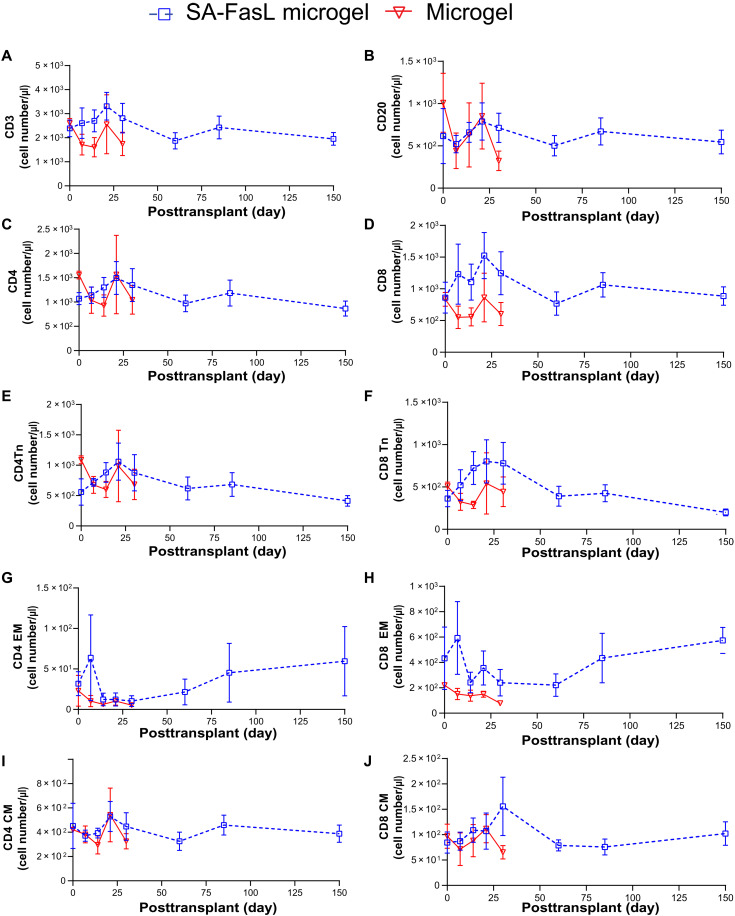
Local delivery of SA-FasL microgels does not alter peripheral blood lymphocyte populations. (**A** to **J**) Numbers of circulating (A) CD3^+^, (B) CD20^+^, (C) CD4^+^, and (D) CD8^+^ cells, as well as naïve (Tn) (E) CD4^+^ and (F) CD8^+^, effector memory (EM) (G) CD4^+^ and (H) CD8^+^, and central memory (CM) (I) CD4^+^ and (J) CD8^+^ lymphocytes in SA-FasL Microgel–treated subjects (dashed blue lines, mean, SEM, *n* = 3 to 4) and Microgel-receiving recipient (red solid lines, mean, SEM, *n* = 3) animals.

Peripheral blood mononuclear cells (PBMCs) were harvested at selected time points, immunostained, and evaluated by flow cytometry using validated gating strategies (fig. S8). Figure 4 presents longitudinal profiles for immune cell populations. For both SA-FasL Microgel and Microgel animals, CD20^+^ B cell as well as CD3^+^, CD4^+^, and CD8^+^ T cell levels remained stable over time, with minor fluctuations among individual animals in both groups (CD20^+^: *P* = 0.5069 and *P* = 0.2531; CD3^+^: *P* = 0.3405 and *P* = 0.4959; CD4^+^: *P* = 0.5079 and *P* = 0.5142; CD8^+^: *P* = 0.2287 and *P* = 0.5277). Similarly, no differences in CD28^+^CD95^−^ CD4^+^ or CD8^+^ naïve, CD28^+^CD95^+^ CD4^+^ or CD8^+^ central memory, and CD28^−^CD95^+^ CD4^+^ or CD8^+^ effector memory T cells were observed over time (CD4^+^ naïve: *P* = 0.4621 and *P* = 0.5102; CD8^+^ naïve: *P* = 0.2524 and *P* = 0.5626; CD4^+^ central memory: *P* = 0.5977 and *P* = 0.4785; CD8^+^ central memory: *P* = 0.4420 and *P* = 0.4790; CD4^+^ effector memory: *P* = 0.4815 and *P* = 0.4829; CD8^+^ effector memory: *P* = 0.2442 and *P* = 0.0844). The lack of differences in these immune cell populations in systemic circulation over time for SA-FasL Microgel animals is expected as we previously showed that the immunomodulatory effects of SA-FasL are localized to the graft (*18*, *19*).

**Fig. 5.**
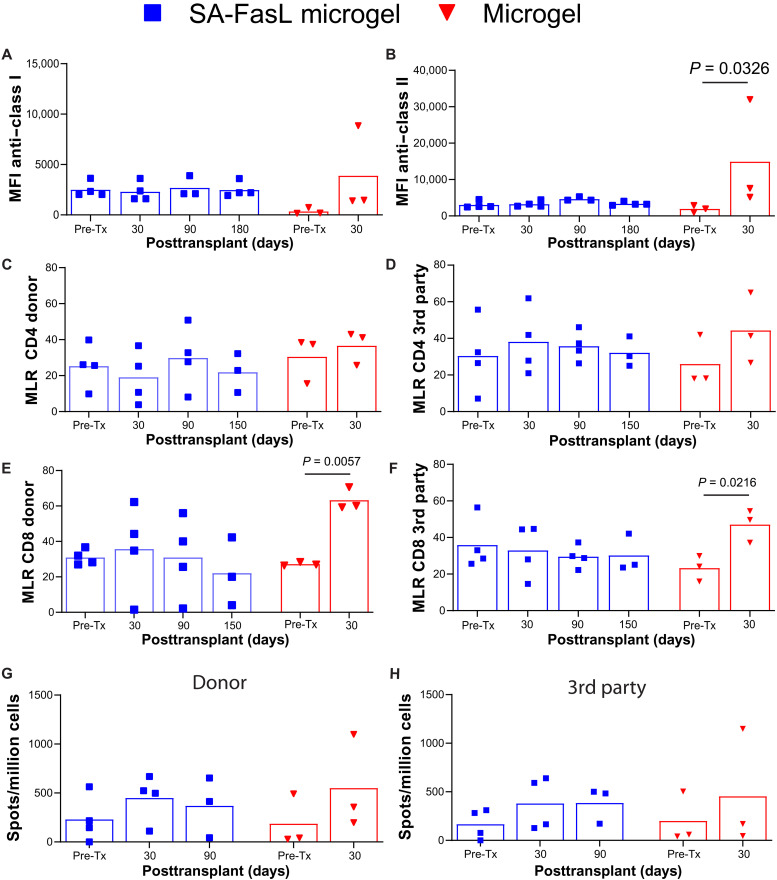
SA-FasL microgel recipients display no significant changes in pre- to posttransplant anti-donor MHC antibodies, CD4^+^ and CD8^+^ T cell proliferative responses to donor and third-party stimulators, and IFN-γ–secreting cell numbers. (**A** and **B**) IgG responses (mean, individual points) to donor-specific MHC (A) class I and (B) class II epitopes for SA-FasL Microgel (blue, *n* = 3 to 4) and Microgel (red, *n* = 3) subjects, demonstrating no donor-specific activation in treated subjects (class I: *P* = 0.2904, class II: *P* = 0.0754). No positive donor-specific antibodies to MHC I were detected in Microgel NHPs (*P* = 0.3292). Nonetheless, these control subjects generated antibodies against donor MHC II (*P* = 0.0326). (**C** to **F**) Mixed lymphocyte reaction (mean, individual points) to CD4^+^ (C) donor and (D) third-party stimulators and CD8^+^ (E) donor and (F) third-party antigens for SA-FasL Microgel (blue, *n* = 3 to 4) and Microgel (red, *n* = 3) subjects showing no differences in responses for the latter (CD4^+^ donor: *P* = 0.4200; CD4^+^ third-party: *P* = 0.6949; CD8^+^ donor: *P* = 0.4145; CD8^+^ third-party: *P* = 0.7858). Microgel animals exhibited no responses against donor (*P* = 0.6082) or third-party (*P* = 0.0555) antigens in CD4^+^ compartment, but CD8^+^ T cell responses were elevated against both donor (*P* = 0.0057) and third-party (*P* = 0.0216) stimulators. (**G** and **H**) ELISpot IFN-γ counts (mean, individual points) for (G) donor and (H) third-party stimulation for the SA-FasL Microgel (blue, *n* = 3 to 4) and Microgel (red, *n* = 3) subjects. No differences in frequency of IFN-γ–secreting cells in circulation between pre- and posttransplant time points for SA-FasL Microgel (donor: *P* = 0.2485; third-party: *P* = 0.1445) or Microgel subjects (donor: *P* = 0.0824; third-party: *P* = 0.1413). Repeated-measures ANOVA was used with pairwise comparisons to pretransplant/day 0 values using Dunnett’s test.

Generation of IgG antibodies against donor MHC antigens was assessed by flow cytometry before and at multiple time points after transplantation. No positive donor-specific antibodies to MHC class I (*P* = 0.2904) or MHC class II (*P* = 0.0758) were detected over time in SA-FasL Microgel subjects (Fig. 5, A and B). In contrast, there was a significant increase in the titers of antibodies to MHC class II (*P* = 0.0326), but not class I (*P* = 0.3292), in the serum of control Microgel NHPs at 1 month after transplantation (Fig. 5, A and B). Mixed lymphocyte reactions against donor and third-party CD4^+^ and CD8^+^ T cells also revealed unchanged responses between pre- and posttransplant conditions for SA-FasL Microgel NHPs (CD4^+^ donor: *P* = 0.4200; CD4^+^ third-party: *P* = 0.6949; CD8^+^ donor: *P* = 0.4145; CD8^+^ third-party: *P* = 0.7858) (Fig. 5, C to F). Although Microgel subjects exhibited unchanged CD4^+^ T cell response against donor (*P* = 0.6082) and third-party (*P* = 0.0555) stimulators, the CD8^+^ T cell compartment showed increased responses toward both donor (*P* = 0.0057) and third-party (*P* = 0.0216) antigens (Fig. 5, C to F). Enzyme-linked immunospot (ELISpot) assays demonstrated no differences in interferon-γ (IFN-γ) secretion between pre- and posttransplant time points for SA-FasL Microgel (donor: *P* = 0.2485; third-party: *P* = 0.1445) or Microgel (donor: *P* = 0.0824; third-party: *P* = 0.1413) subjects (Fig. 5, G and H). Multiplexed immunobead assays showed no significant differences between pre- and posttransplantation levels of proinflammatory and anti-inflammatory cytokines and chemokines in the serum of SA-FasL Microgel and Microgel subjects (fig. S9). Last, antibodies against SA-FasL protein were not detected in the serum of SA-FasL Microgel animals at early time points but developed at ~2 weeks after transplantation (fig. S10, A to D). Most antibodies were against the SA domain of the SA-FasL protein (fig. S10E). These results show that antibodies against SA-FasL develop ~2 weeks after transplantation, when SA-FasL is undetectable systemically, and primarily are against the SA moiety of the chimeric protein. The presence of such antibodies did not negatively affect the efficacy of our protocol in sustaining allogeneic islet graft survival.

## DISCUSSION

Achieving indefinite survival of allogeneic grafts without chronic immunosuppression remains an elusive goal in clinical transplantation. For islet transplantation in particular, a reliable and safe tolerogenic regimen could expand the therapeutic application of islet transplantation for T1D given the delicate risk/benefit considerations balancing restoration of β cell function with immunosuppression toxicities (*28*–*32*). Here, we demonstrate that SA-FasL–presenting microgels cotransplanted with allogeneic islets are effective in sustaining long-term (>6 months) survival and excellent glycemic control in diabetic NHPs without chronic immunosuppression. These subjects demonstrated glucose-responsive insulin secretion and C-peptide levels comparable to the prediabetic state. In marked contrast, control animals cotransplanted with microgels without SA-FasL rejected the islet graft acutely. Sustained survival was associated with an increased number of FoxP3^+^ (a marker of T_regs_) cells at the graft site without a detectable change in systemic frequency of various immune cell populations. The pre- and posttransplant proliferative responses of CD4^+^ and CD8^+^ T cells to donor and third-party stimulators were equivalent for subjects that received SA-FasL–presenting microgels, indicating the lack of systemic unresponsiveness. In addition, NHPs receiving SA-FasL–presenting microgels did not reveal changes between pre- and posttransplant IFN-γ–secreting cell numbers and anti-donor MHC antibodies. In contrast, subjects that received control microgels generated antibodies against donor MHC molecules and exhibited increased CD8^+^ T cell proliferative responses to donor and third-party antigens. Increased numbers of FoxP3^+^ cells in the graft site and unchanged systemic T cell frequency and responses to donor antigens are consistent with our studies in rodents, demonstrating that SA-FasL confers localized tolerance to the graft that requires T_regs_ (*18*, *19*, *33*). Our results are consistent with prior work demonstrating the role of T_regs_ in “induced” immune privilege to allografts (*34*, *35*). T_regs_ were shown to protect allogeneic hematopoietic stem cells in naïve recipients by colocalizing in the marrow cavity (*34*), and immune privilege mediated by T_regs_ was also shown for skin and lung allografts (*36*, *37*). T_regs_ manifest their suppressive function by various means, including converting T effector cells into T_regs_ (*38*), altering the function of dendritic cells within draining lymph nodes (*39*), and expression of various immunoregulatory cytokines and chemokines (*40*, *41*). Last, subjects receiving allogeneic islets and SA-FasL microgels exhibited normal liver and kidney metabolic function and organ histology and continued to gain weight, indicating that the immunomodulatory biomaterial has an acceptable safety profile. We did not observe any of the reported FasL toxicity (*42*–*44*), which we attribute to the form and local presentation of SA-FasL.

Although diabetic NHPs receiving allogeneic islets co-transplanted with SA-FasL microgels displayed long-term islet acceptance and normal fasting glycemic control, they did not achieve insulin independence. Nevertheless, there was 80 to 90% reduction in EIR as compared with the pretransplant diabetic levels to control postprandial blood glucose. The idea that insulin independence was not achieved is not unexpected as NHP allogeneic islet transplantation is complex and demanding, and single-donor islet transplantation (as done in this study) generally represents a marginal islet mass. Similarly, islet mass from a single donor is generally insufficient to restore full euglycemia in human islet transplantation. The Clinical Islet Transplant Consortium registration trials for islet alone and islet after kidney transplantation found that only ~20% of patients were rendered normoglycemic after single human donor islet transplantation (*45*). The same issue applies to allo-islet NHP transplantation. The sustained C-peptide levels, improved glycemic control, and results of the periodic IVGTT document stable and robust islet function. Notably, we did observe gradual improvement in islet graft function in three of four recipients of allogeneic islets cotransplanted with SA-FasL microgels, and one recipient almost achieved stable insulin independence toward the end of the study, indicating the efficacy and stability of the protocol in controlling chronic immune responses to the graft.

MHC typing demonstrated that all donor-recipient pairs were overall incompatible. However, for both SA-FasL Microgel and control cohorts, each group has only one pair of animals that were MHC fully mismatched, whereas all other pairs were either Mafa-A or Mafa-B haploidentical or MHC haploidentical due to limited animal selection to achieve MHC full mismatch. Although the IVGTT data for the fully mismatched recipient M518 at 6 months after transplantation showed glucose disposal better than when it was naïve, this animal and the fully mismatched recipient M517 in the control group showed less robust nonfasting blood glucose control after transplantation compared to their peers in their respective groups. This poses the question of whether the ratio of SA-FasL–presenting microgels to islets may need to be modified in the setting of full MHC mismatch. We know that islet β cells do not express class II antigens under normal physiological conditions and hence lack direct recognition via CD4^+^ T cells (*46*, *47*). This might be one of the reasons why current clinical islet transplantation does not practice MHC matching and the newly completed two phase 3 Clinical Islet Transplantation Consortium clinical trials (*45*, *48*) did not provide insights in this regard either. Many factors, such as animal eating habits, pretransplant insulin requirement, the quality and quantity of islet transplanted, baseline donor specific anti-HLA antibodies, and others, all contribute to the posttransplantation metabolic results in our NHP model, but the MHC matching versus metabolic results deserves future study. Furthermore, even with MHC fully matched, grafts will be rejected because of minor antigens in the NHP allogeneic transplant model. However, the impact of the SA-FasL microgels is still clearly evident because two of the control animals were MHC haploidentical but still rejected the grafts at ~1 month after transplantation.

Immunological reaction to transplanted allogeneic islets involves both allo- and autoreactive pathways. Both auto- and alloreactive T cells express Fas on their surface after antigen recognition and activation and become sensitive to apoptosis induced by the Fas/FasL interaction. Therefore, FasL has the potential for use as an immunomodulator to eliminate these T effectors in the setting of islet transplantation. Our strategy is to deliver FasL locally within the graft microenvironment in a controlled and sustained fashion using advanced synthetic materials. This is a unique advantage over FasL gene therapy, because uncontrolled, continuous expression of FasL, which has pleiotropic activity and various modes of expression that may be differentially regulated by target tissues, may have unintended consequences. Ectopic expression of FasL using gene therapy for immunomodulation has resulted in mixed outcomes, with some studies showing a detrimental impact of FasL expression on graft survival (*49*). The localized presentation of SA-FasL using microgels also overcomes complications associated with ectopic expression of FasL in target tissues using gene therapy and potential toxicities as reported for agonistic antibodies to Fas (*50*). Last, SA-FasL–engineered microgels provide the flexibility of an off-the-shelf product for wider clinical applications, as these immunomodulatory materials can be prepared at the time of transplantation and simply admixed with islets for delivery without the need of encapsulating or chemically modifying islets to present proteins.

We note limitations of the present study. Because of logistical, ethical, and cost considerations, NHP allogeneic islet transplantation studies comprise a small number of subjects, limiting statistically comparisons, and the results may not be representative of a larger cohort of subjects. Furthermore, although the STZ-induced NHP diabetes model provides a rigorous test of allogeneic islet transplantation, it does not involve autoimmunity, an important consideration when transplanting in the setting of human T1D. Last, the omentum of a small primate is a thin gossamer-like vascularized membrane that is not fat laden and thick. The human omentum is often dissimilar to that of the primate. Although initial studies support omentum as an alternative to intraportal transplantation in humans (*23*), additional studies are necessary to establish the validity of this site for clinical islet transplantation. In this study, we decided against transplanting SA-FasL–presenting microgels and islets to the liver as currently done clinically over safety concerns that the additional volume of the microgels could increase the risk of portal vein thrombosis. Further studies in NHP portal transplantation modes or modification of the microgel delivery vehicle to increase compatibility with the portal vein route are necessary. In addition to the omental site, the recent report of using intrapleural space as a site for transplantation of allogeneic pancreatic islets (*26*) is of interest and could potentially be combined with this strategy.

## MATERIALS AND METHODS

### Animals and pairing selection

Study protocols were approved by the Institutional Animal Care and Use Committee at the Massachusetts General Hospital Research Institute. Naïve, captive-bred, male or female cynomolgus monkeys (*Macaca fascicularis*) were purchased from Charles River Laboratory (Houston, TX). All animals were certified under the standards of U.S. Interstate and International Certificate of the Health Examination for Small Animals. Bacteriologic and serological tests were performed on each animal to ensure that they were not infected with parasites, Salmonella/shigella or Tuberculosis bacteria, Simian T-cell leukemia viruses, Simian immunodeficiency virus, herpes B, and Schmidt-Ruppin virus before being transported to Massachusetts General Hospital Center for Comparative Medicine and Laboratory Animal Services (CCM) for housing. NHPs were fed three times daily (7:00 a.m., 11:00 a.m., and 2:00 p.m.) with certified primate food (LabDiet, catalog no. 5038) and supplemented with fresh fruit daily (9:00 a.m. and 3:30 p.m.).

After an 8-week quarantine period at CCM, blood was drawn for ABO typing, MHC genotyping analysis (AIDS Vaccine Research Labs, University of Wisconsin, Madison, WI), and T cell subset analysis before transplantation. Recipients also underwent a 2- to 4-month training to cooperate with procedures such as drug administration, hand feeding, and voluntary presentation of their tails to facilitate blood draw for blood glucose measurement. Donors and recipients were paired on the basis of ABO blood group compatibility and MHC mismatching (tables S1 and S2). In each case, islets from one donor were transplanted into one recipient.

### Diabetes induction and management

NHPs were fasted overnight before administration of STZ at a dose of 75 mg/kg (Zanosar, Teva Parenteral Medicines, Irvine, CA). Sterile STZ powder was diluted in 5.0 ml of 0.9% NaCl and administered through the saphenous vein over 2 min followed by infusion of 30 ml of NaCl. Starting the next day, blood glucose levels were monitored twice daily (8:00 to 9:30 a.m. and 3:30 to 5:00 p.m.) via tail pricking (Accu-check Aviva, Roche Diagnostics, Indianapolis, IN). STZ-induced diabetes was defined as three consecutive fasting glucose level readings of >300 mg/dl and C-peptide levels of <0.5 ng/ml [Mercodia C-peptide enzyme-linked immunosorbent assay (ELISA), Mercodia AB, Uppsala, Sweden]. Post-STZ period is defined from the time of STZ injection until the day of islet transplantation. Posttransplant graft rejection was defined as three consecutive fasting blood glucose readings of >180 mg/dl or nonfasting blood glucose of >250 mg/dl. Exogenous insulin (Humulin R, Lilly, Indianapolis, IN) and Lantus (Lilly, Indianapolis, IN) were administered on a sliding scale regimen to achieve a postprandial blood glucose level of <250 mg/dl before transplant or after graft rejection was defined. Animals having induced diabetes for at least 12 days were used as recipients of islet transplantation.

### Treatment regimens

For all recipient animals, the day of allogeneic islet transplant is defined as day 0. Animals received rapamycin (LC Laboratories, Woburn, MA) at a dose of 0.2 mg/kg intramuscularly daily for a total of 3 months starting on day −3, with target blood trough level of 40 ng/ml for the first 2 weeks and then 20 ng/ml until the 3-month time point after transplant, when rapamycin was discontinued without weaning. Subjects received islets and SA-FasL–presenting PEG microgels or control microgels at a 1 IEQ:2 PEG microgel ratio. After transplantation, daily low-dose exogenous insulin (two to four units) was administrated to all recipients for the first 28 days to promote islet engraftment by allowing islet “rest.” Graft recipients were also subjected to prophylactic ganciclovir treatment for cytomegalovirus.

### Donor pancreatectomy

Donor pancreatectomy was performed on the same day as islet transplantation. In brief, a median sternotomy with a midline abdominal incision was performed. Once a 12-gauge cannula connected to infusion set of hypothermic University of Wisconsin (UW) organ preservation solution was placed into the aorta at the renal artery level, 2000 U of heparin (SAGENT Pharmaceuticals, Schaumburg, IL) was given intravenously. Subsequently, the abdominal aorta was cross clamped at the subdiaphragmatic level. Lesser sac was packed with iced saline, while 1000 ml of UW solution was delivered from the aorta cannula for perfusion. The inferior vena cava was transected to provide proper venous drainage during perfusion. The entire pancreas was mobilized and excised subsequently.

### Islet isolation and transplantation

The protocol of islet isolation was based on a modified human islet isolation protocol as we previously reported (*7*, *51*). Briefly, the pancreas was enzymatically digested using purified Thermolysin and Collagenase blend (Vitacyte, Indianapolis, IN). Islets were purified from the digestion using continuous OptiPrep gradient (densities of 1.11 to 1.06) and a COBE 2991 blood cell processor (Gambro BCT Inc., Lakewood, CO) to separate islet from exocrine tissue. Samples taken from different fractions after purification were used to assess the purity of each cell fraction. Only fractions with >50% purity by dithizone stain were combined for transplantation. Final islet preparations were enumerated by manual counting, sizing, and converting islet particle number to IEQs based on a 150-mm diameter. Islets were then put into 15 ml of CMRL 1066 transplant media (Cellgro, Manassas, VA), supplemented with 10% human serum albumin (Grifols Therapeutics, Research Triangle Park, NC) and heparin 70 units/kg recipient body weight ready for transplantation.

Under general anesthesia, a small midline abdominal incision was performed. The omentum was mobilized and draped over on a sterile moist towel with as minimal manipulation as possible. Islets were resuspended with PEG microgels with or without SA-FasL protein at a 1:2 ratio of IEQ to microgel in a minimal volume of NHP autologous plasma and carefully dripped onto the omentum surface. The islets were immobilized on the omentum by topical recombinant thrombin (Recothrom, ZymoGenetics) layered over the islet slurry, followed by another layer of autologous plasma to create a degradable biologic fibrin matrix (*23*). The omentum was then folded onto itself and held in place by the thrombin-induced fibrin glue to form a microenvironment (omental pouch) to host the islets and SA-FasL microgels.

### Intravenous glucose tolerance test

Animals were sedated with ketamine hydrochloride after overnight fasting (no insulin was administered the night before). Catheters (22-gauge) were placed into bilateral saphenous veins for glucose delivery and blood sampling separately. Fifty percent of dextrose (Hospira Inc., Lake Forest, IL) solution (0.5 g/kg) was injected over a 30-s period at *t* = 0. Blood samples were taken before dextrose injection and at 1, 3, 5, 10, 15, 20, 30, 60, and 90 min after injection to measure glucose. Serum samples were frozen at −80°C for subsequent analysis. Insulin and C-peptide were measured in duplicate with an ELISA kit (Mercodia AB, Uppsala, Sweden, catalog nos. 10-1132-01 and 10-1141-01).

### SA-FasL protein production

The SA-FasL protein was produced using a Drosophila Expression System expression system and our established protocols (*18*, *52*). Briefly, S2 cells stably transfected with the SA-FasL construct were induced for protein expression using CuSO_4_. Culture supernatant was collected 72 hours after induction and subjected to protein purification using a FLAG-tagged immunoaffinity purification system. The purified protein was analyzed by SDS–polyacrylamide gel electrophoresis for purity, assessed for concentration using bicinchoninic acid, and tested for the presence of endotoxin using limulus amebocyte lysate endpoint assay (Cambrex Biosciences). The apoptotic function of SA-FasL protein was assessed using the mouse A20 B cell lymphoma line constitutively expressing Fas receptor as an in vitro potency assay. Protein lots having EC_50_ (median effective concentration) apoptotic activity of 5 to 20 ng/ml were then assessed for biotin binding capacity using mouse splenocytes surface-modified with biotin and analyzed using flow cytometry.

### SA-FasL–presenting microgels

PEG microgels functionalized with biotin were prepared as described (*19*, *53*). Briefly, poly(dimethyl siloxane) microfluidic flow focusing devices with 200-μm nozzles were cast and assembled. Syringe pumps were used to infuse polymer and cross-linking solutions at a continuous rate such that the flow focusing geometry of the microfluidic device generates polymer droplets. PEG-biotin solution (5% 20 kDa PEG-4MAL reacted with 2.0 mM biotin-PEG-thiol) was infused into the microfluidic device and focused into 160- to 180-μm-diameter droplets by coflowing with shielding solution consisting of light mineral oil and 2% SPAN80 surfactant. The droplets were then cross-linked by introduction into a flow stream of a dithiothreitol [30 mg/ml in phosphate-buffered saline (PBS)] emulsion in mineral oil at a ratio of 1:15. After rapid cross-linking, microgels were collected in a solution of PBS supplemented with 1% human serum albumin. To remove excess oil and crosslinking solution, microgels were washed five times in PBS with 1% human serum albumin by centrifugation. After the final wash, capsules were stored in PBS with 1% human serum albumin at 4°C for up to 2 weeks. To tether SA-FasL onto biotin-functionalized microgels, SA-FasL was incubated with biotin microgels in PBS with gentle rocking for 30 min (1 μg of SA-FasL/1000 microgels) and washed in PBS with 1% human serum albumin. Previously fabricated PEG microgels were functionalized with SA-FasL on the day of transplant.

### Histology

Samples from liver, spleen, intestine, lymphoid, lung, heart, omentum, and native pancreatic tissue were fixed in 10% formaldehyde before being processed and embedded in paraffin. Serial sections of 5 μm thickness were cut. H&E staining was performed for routine histology. Immunohistochemistry was conducted using anti-FoxP3 antibody (eBioscience, San Diego, CA). Biotinylated horseradish peroxidase (HRP) or fluorescently labeled secondary antibodies were used, and for HRP-conjugated antibodies, signals were detected by diaminobenzidine (Vector Labs, Burlingame, CA). Slides were counterstained with hematoxylin or 4′,6-diamidino-2-phenylindole (DAPI) and mounted.

### Preparation of PBMCs

PBMCs were isolated from whole heparinized blood using a Percoll density gradient method. Briefly, 10 ml of blood was transferred to a 50-ml conical tube prefilled with 35-ml sterile saline solution. Next, 10 ml of 60% Percoll gradient (Sigma-Aldrich, St. Louis, MO) was pipetted to underlay the diluted blood. The layered solution was centrifuged at 700*g* for 30 min at room temperature, with the brake turned off. The PBMC-rich buffy layer was harvested, and contaminating red blood cells were removed by standard water shock treatment. PBMCs were washed three times with 1× PBS, and viable cells were counted by trypan blue exclusion. PBMCs were either frozen at −80° and then stored in liquid nitrogen or used immediately for assays.

### Flow cytometric analyses

Fresh blood cells (<6 hours) were labeled with fluorescein isothiocyanate (FITC)–, phycoerythrin (PE)–, peridin chlorophyll protein (PerCP)–, or allophycocyanin (APC)–conjugated antibodies including CD3, CD4, CD8, CD20, CD95, CD28, or immunoglobulin M (IgM) (BD Pharmingen, San Jose, CA). FACSVerse flow cytometer (Becton Dickinson, San Jose, CA) and FlowJo software (TreeStar Inc., Ashland, OR) were used for immune cell subset analysis.

### Mixed lymphocyte reaction

T cells from recipient PBMCs were purified using the Pan T Cell Isolation Kit (catalog no. 130-091-993, Miltenyi Biotec) as responder cells to perform mixed lymphocyte reactions. Responder cells were labeled with carboxyfluorescein succinimidyl ester (CFSE) (Life Technologies, Carlsbad, CA) at a concentration of 5 μM per 10^7^ cells at room temperature in the dark for 5 min and then plated at a concentration of 3 × 10^5^ cells per well in 196-well U-bottom plates for coculture with irradiated (3000 cGy) VPD-450–labeled stimulator PBMCs from donor or third-party NHPs. PBMCs from the responder animal were used as negative controls, and phytohemagglutinin (PHA) was used for positive controls. After 5 days in coculture at 37°C, 5% CO_2_, the cells were stained with antibodies desired and CFSE dilution was assessed by flow cytometry.

### Detection of anti-donor alloantibody

Donor PBMCs were first incubated with recipient serum for 30 min at 4°C, followed by Alexa Fluor 488–conjugated goat anti-human IgG antibody (Jackson ImmunoResearch, West Grove, PA) for 30 min at 4°C. PBMCs were costained with PerCP-conjugated anti-CD20 and IgM APC anti-human antibodies (BD Pharmingen, San Jose, CA) for 30 min at 4°C. After washing, cells were fixed with 2% paraformaldehyde (Santa Cruz Biotechnology, Santa Cruz, CA) and analyzed with a BD FACSVerse flow cytometer. A stored mixture serum from more than four previously rejecting monkeys was used as positive control. A positive reaction of the T and B cells was defined as a shift greater than pretransplant serum control.

### IFN-γ ELISpot assay

ELISpot plates (Millipore, Bedford, MA) were precoated with capture antibody (5 μg/ml) against IFN-γ (Mabtech, Cincinnati, OH) in 1× PBS and stored overnight at 4°C. Plates were washed five times in PBS and blocked with RPMI 1640 (Cellgro, Manassas, VA) containing 10% bovine serum albumin (Sigma-Aldrich) for 1 hour at 37°C in a humidified incubator followed by three washes in PBS. Responding PBMCs (3.0 × 10^5^ cells) were added to each well in 100 μl of complete RPMI 1640 supplemented with 10% pooled naïve monkey serum and l-glutamine, penicillin/streptomycin, and Hepes buffer (Invitrogen). The responding cells were cultured with an equal number of irradiated stimulating cells, medium alone, or PHA at 1 μg/ml (Sigma-Aldrich). After 48-hour incubation at 37°C, the plates were washed and biotinylated detection antibodies against IFN-γ (Mabtech, Cincinnati, OH) were added. The plates were then incubated at room temperature for 2 hours, washed with PBS, and incubated with SA-HRP (Mabtech) in PBS for 1 hour at room temperature, followed by an additional five washes. Trimethylboron (TMB; Moss, Pasadena, MD) was used to develop the spots. Development was done until distinct spots appeared. Plates were analyzed with an ELISpot image analyzer (Cellular Technology, Cleveland, OH).

### Cytokine Luminex assay

A monkey cytokine magnetic 29-Plex panel (catalog no. LPC0005M, Invitrogen) was used to measure the concentration of cytokines in the plasma. Samples were diluted 1:2 in assay diluent and then added to the wells. In brief, 25 μl of 1× antibody beads was added into each assay well followed by 50 μl of incubation buffer. Diluted standards (100 μl) were added into standard wells, and 50 μl of assay diluent followed by 50 μl of sample was added into sample wells. The plate was covered and incubated for 2 hours at room temperature on an orbital plate shaker. Then, the plate was washed twice with 1× wash solution. Biotinylated detector antibody (100 μl) was added to all assay wells and incubated for 1 hour at room temperature on a shaker. After washing twice with wash solution, 100 μl of SA-R-phycoerythrin solution was added to each assay well and incubated for 30 min at room temperature on a shaker. The plate was washed three times with 200 μl of wash solution. Wash solution (150 μl) was added to each assay well, and then the plate was inserted into the XY platform of the MAGPIX instrument (Luminex XMAP Technology) and analyzed. The concentration of samples was determined from the standard curve using a curve fitting software.

### Detection of SA-FasL protein and anti-SA and anti-FasL antibody

The presence and quantity of SA-FasL protein in the plasma of SA-FasL microgel–treated NHPs was assessed using a rat FasL sandwich ELISA kit from RayBiotech, with a sensitivity of 90 pg/ml. Briefly, serum from before and various time points after transplant obtained from NHPs receiving SA-FasL microgels were used per manufacturer’s instructions. SA-FasL in plasma samples was quantified using a standard curve and reported as nanograms per milliliter.

The titers of anti–SA-FasL antibodies in NHPs receiving SA-FasL microgels were determined using a standard ELISA. Briefly, Costar 96-well titer plates (Corning Life Sciences) were coated with SA-FasL protein (1 μg/ml) in PBS overnight at 4°C. Plates were washed twice with PBS containing 0.5% Tween 20, and wells were incubated with the blocking buffer (PBS containing 5% dry milk + 0.5% Tween 20) overnight at 4°C. Plates were then washed twice with PBS containing 0.5% Tween 20. Fifty microliters of twofold serial dilutions of serum that had been preincubated with the blocking buffer for 30 min was added to each well in duplicate and incubated for 90 min at room temperature. Wells were then washed two times with PBS + 0.5% Tween 20 and incubated for 1 hour at room temperature with goat anti-monkey IgG-HRP antibody (Thermo Fisher Scientific, Waltham, MA) diluted in 1:20,000 in the blocking buffer. Wells were washed twice with PBS + 0.5% Tween 20 and incubated with 50 μl of TMB substrate (BD Biosciences) for 30 min, and the reaction was stopped with 2 N sulfuric acid. Absorbance was measured at 450 nm, and anti–SA-FasL antibodies were reported as log_10_ titers of the greatest serial dilution with a mean OD_450_ (optical density at 450 nm) value >2-fold the OD_450_ value of naïve serum. Samples with an antibody titer of log_10_ 1.4 or less were considered negative. The titers of antibodies against SA protein were determined using the same scheme for SA-FasL, except that the 96-well titer plates were coated with the SA protein.

### Statistical analyses

Data are reported as mean ± SEM. All analyses were performed using Prism (version 8.4, GraphPad Software). Graft survival between groups was compared using the log-rank (Mantel-Cox) test and Gehan-Breslow-Wilcoxon statistics. Normally distributed data that presented equal variances were analyzed via analysis of variance (ANOVA; unpaired *t* tests for two groups) or log-transformed if the variances were not equal. Dunn’s tests were used for post hoc pairwise comparisons. For longitudinal data, repeated-measures ANOVA was used with pairwise comparisons to pretransplant/day 0 values using Dunnett’s test. *P* values less than 0.05 were considered statistically significant.

## Notes

View/request a protocol for this paper from *Bio-protocol*.
