# Supplementary material for: FasL microgels induce immune acceptance of islet allografts in nonhuman primates
Source: Sci Adv. 2022 May 13;8(19):eabm9881. doi: 10.1126/sciadv.abm9881 (PMC9106299; doi:10.1126/sciadv.abm9881)
Supplement: Supplementary file 1 — Figs. S1 to S10 Tables S1 and S2 [file sciadv.abm9881_sm.pdf]

Science Advances
AAAS

Supplementary Materials for

FasL microgels induce immune acceptance of islet allografts in nonhuman primates

Ji Lei*, María M. Coronel, Esma S. Yolcu, Hongping Deng,
Orlando Grimany-Nuno, Michael D. Hunckler, Vahap Ulker, Zhihong Yang, Kang M. Lee,
Alexander Zhang, Hao Luo, Cole W. Peters, Zhongliang Zou, Tao Chen, Zhenjuan Wang,
Colleen S. McCoy, Ivy A. Rosales, James F. Markmann*, Haval Shirwan*, Andrés J. García*

*Corresponding author. Email: jlei2@mgh.harvard.edu (J.L.); jmarkmann@mgh.harvard.edu (J.F.M.); haval.shirwan@health.missouri.edu (H.S.); andres.garcia@me.gatech.edu (A.J.G.)

Published 13 May 2022, *Sci. Adv.* **8**, eabm9881 (2022)
DOI: 10.1126/sciadv.abm9881

This PDF file includes:

Figs. S1 to S10
Tables S1 and S2

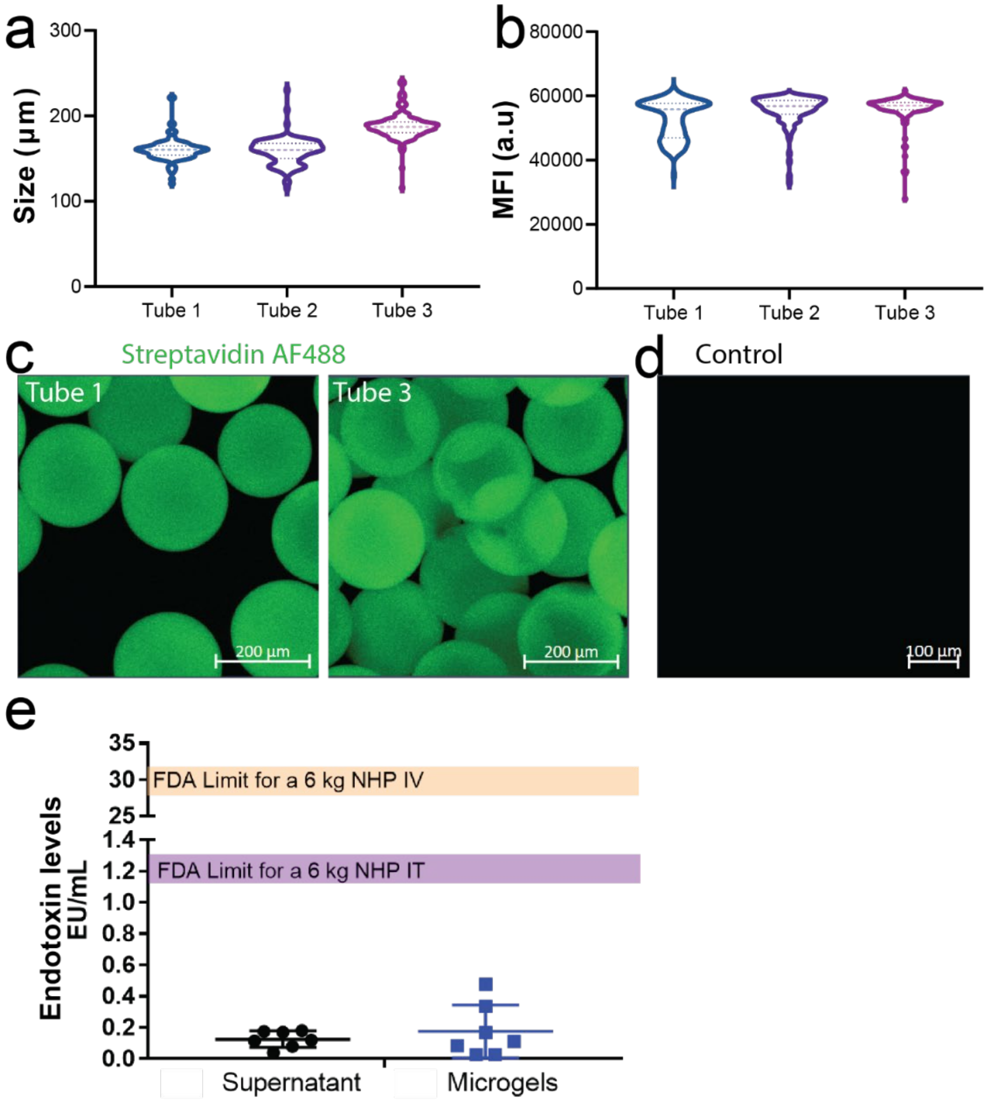

Fig. S1. Quality control of synthetic biotinylated microgels generated by microfluidics polymerization. a, Equivalent size distribution after multiple fabrication runs (violin plots, tube 1: n=97; tube 2: n=102; tube 3: n=159). **b**, Mean fluorescence intensity for AlexaFluor 488-conjuaged streptavidin captured onto biotinylated microgels (violin plots, tube 1: n=97; tube 2: n=102; tube 3: n=159). **c-d**, Confocal microscope images of (**c**) biotinylated and (**d**) control microgels incubated in AlexaFluor 488-conjuaged streptavidin (bar 200 µm and 100 µm, respectively). **e**, Endotoxin levels (mean, SEM) for supernatant (black dots) of microgels and microgels (blue squares). FDA endotoxin limits for intravenous (IV) and intrathecal (IT) delivery for a 6 kg NHP.

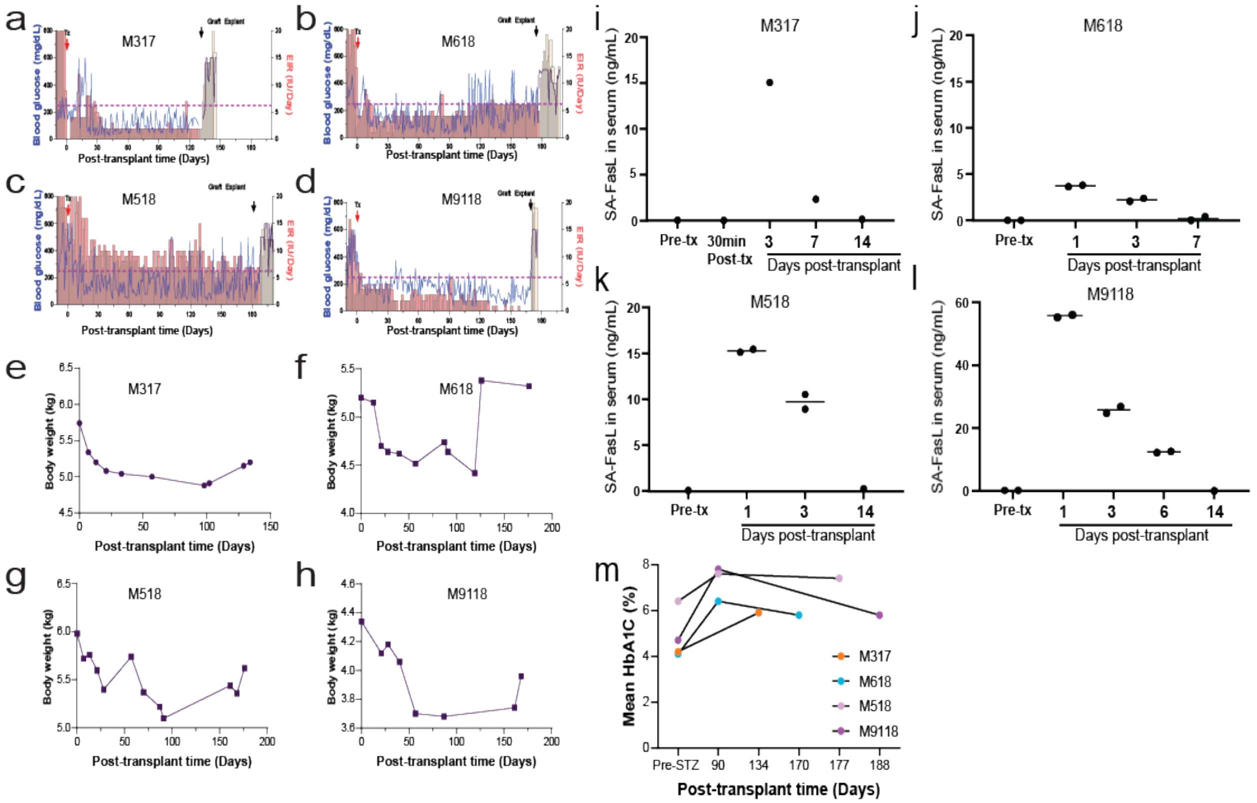

Fig. S2. Glycemic control, body weight, and serum SA-FasL levels for individual NHPs receiving SA-FasL-microgels. a-d, Non-fasting blood glucose levels (blue line; left axis) and daily total external insulin requirement (EIR) (red bars; right axis) for all SA-FasL Microgel receiving subjects. Animals exhibited high blood glucose levels and external insulin demand after STZ treatment prior to transplant. After co-transplantation of islets and SA-FasL-microgels (Tx), animals rapidly became normoglycemic and had significantly reduced external insulin requirement. Animals reverted to hyperglycemic state after graft removal (blood glucose levels, purple lines, left axis; total external insulin requirement, tan bars, right axis). **e-h**, Longitudinal tracking of body weight. **i-l,** Serum levels of SA-FasL in all subjects, reaching background levels between days 7-14 post-transplantation. **m,** Glycated hemoglobin (HbA1c) levels are well controlled during the study course.

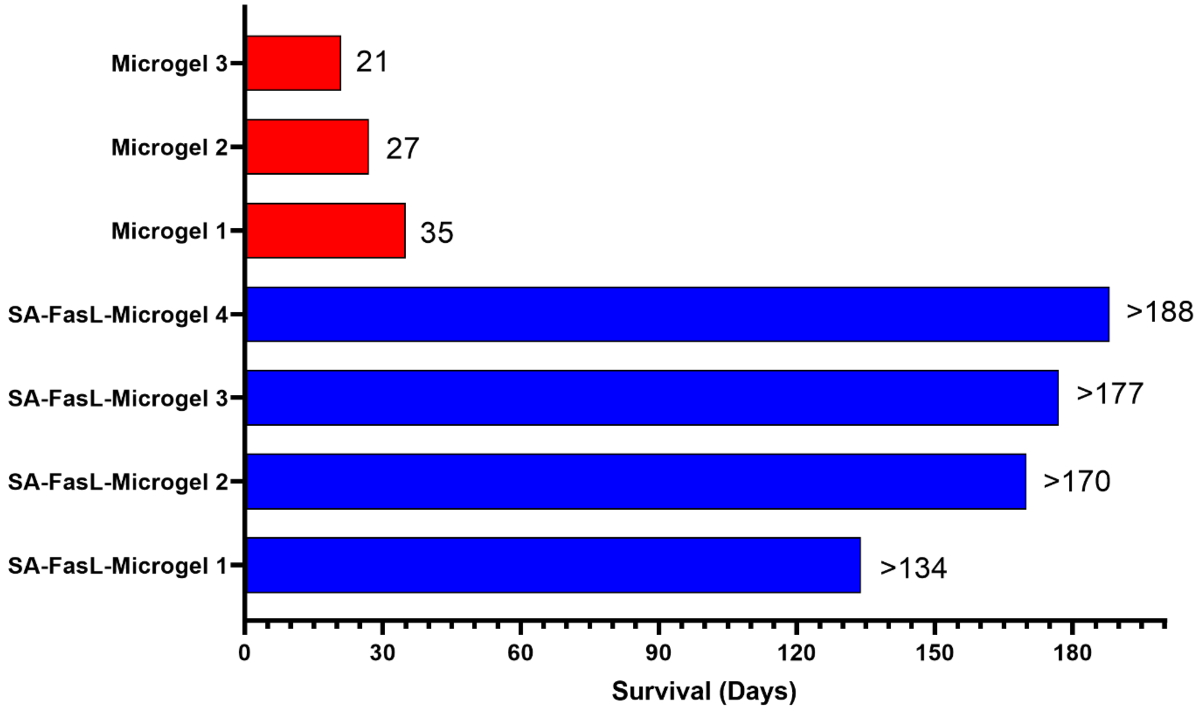

Fig. S3. Islet graft survival for individual recipients. SA-FasL Microgel subjects were terminated on the indicated days in the graph while islet grafts were functioning due to COVID-19 imposed logistical constraints.

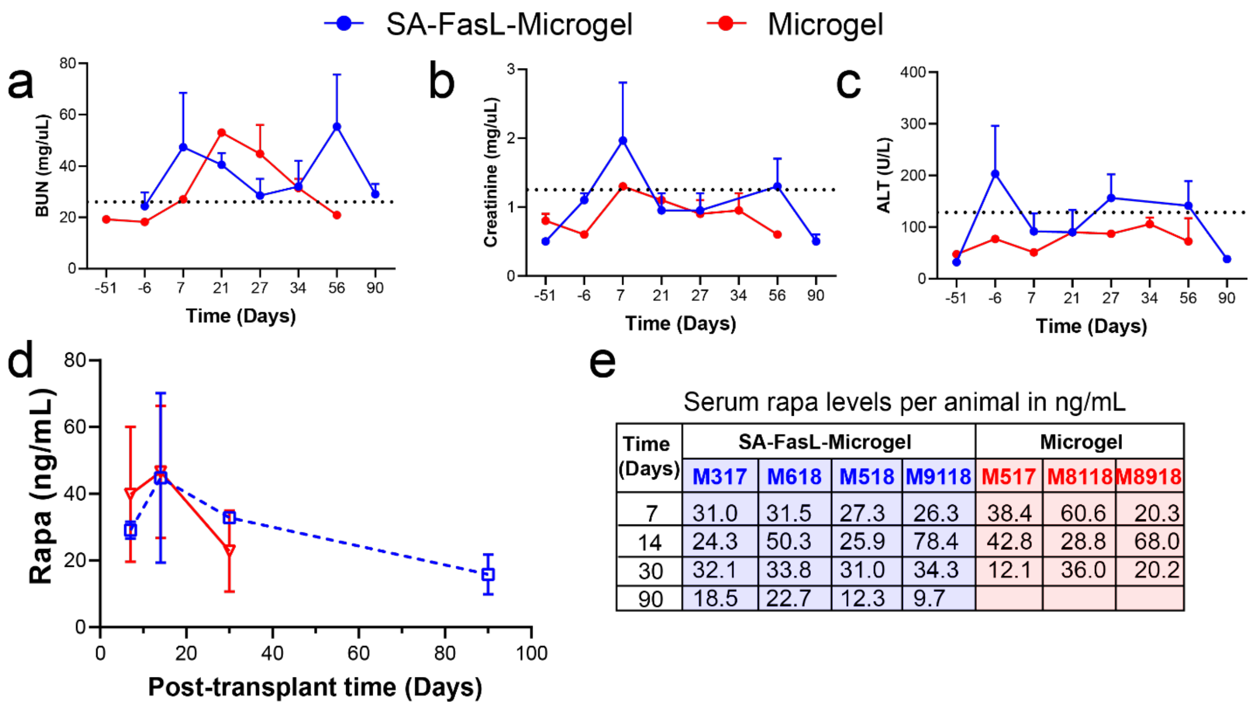

Serum rapa levels per animal in ng/mL

Time (Days)	SA-FasL-Microgel				Microgel		
	M317	M618	M518	M9118	M517	M8118	M8918
7	31.0	31.5	27.3	26.3	38.4	60.6	20.3
14	24.3	50.3	25.9	78.4	42.8	28.8	68.0
30	32.1	33.8	31.0	34.3	12.1	36.0	20.2
90	18.5	22.7	12.3	9.7			

Fig. S4. Serum biomarkers for liver and kidney function and rapamycin serum levels in NHPs receiving SA-FasL or control microgels. a-c, Longitudinal tracking of (**a**) blood urea nitrogen (BUN), (**b**) creatinine, and (**c**) Alanine Aminotransferase (ALT) levels (mean, SEM) in animals receiving SA-FasL Microgels (blue, n=4), and control Microgels (red, n=3). **d,** Longitudinal tracking of rapamycin concentration (mean, SEM) in serum demonstrates no differences in rapamycin levels between subjects of SA-FasL Microgels (dashed blue lines, n=4) and control Microgels (red lines, n=3) subjects (two-tailed Mann-Whitney test, p=0.63). **e**, Serum rapamycin levels for individual recipients.

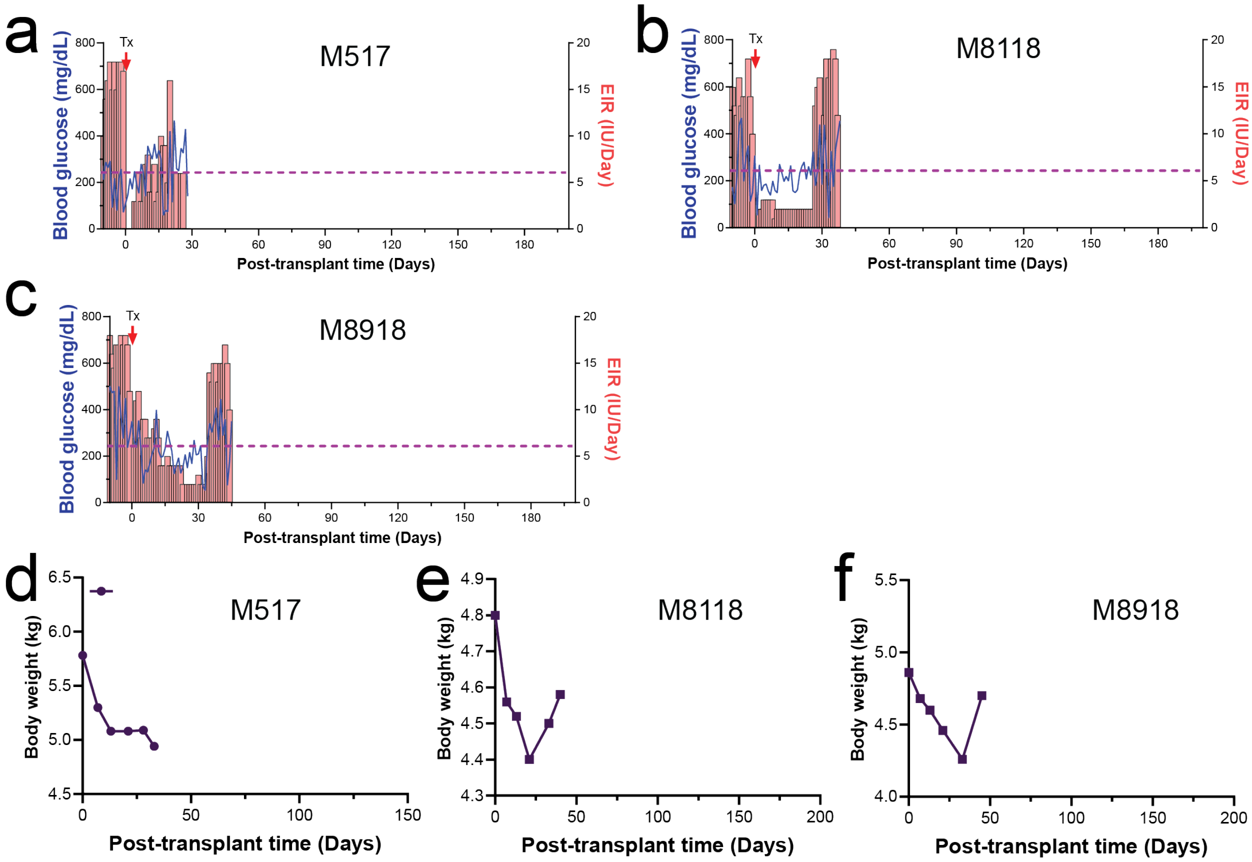

Fig. S5. Non-fasting blood glucose levels and body weight for individual NHPs receiving control microgels. a-c, Non-fasting blood glucose levels (blue line; left axis) and daily total external insulin requirement (red bars; right axis) for all subjects. Animals exhibited high blood glucose levels and external insulin demand prior to transplant. After restoring normoglycemia following transplantation (Tx), animals became hyperglycemic and required higher external insulin around 1 month post-transplantation. **d-f**, Longitudinal weight dynamics post-transplant.

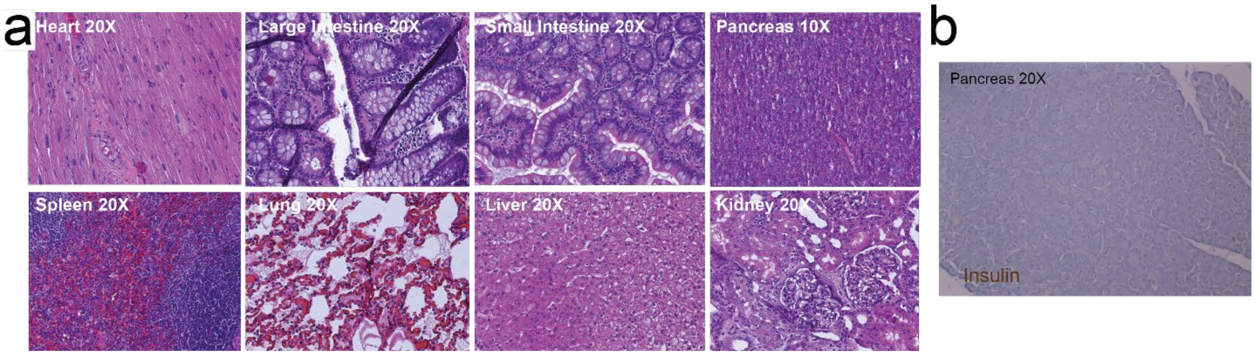

Fig. S6. Histological analyses of tissues from animals receiving SA-FasL-microgels showing no signs of toxicity. a, Histopathological examination of various organs demonstrates no toxicity to SA-FasL-microgel transplantation in omentum. Images are representative of treated animals. **b**, Negative insulin staining in pancreas at necropsy at 6 months post-transplant.

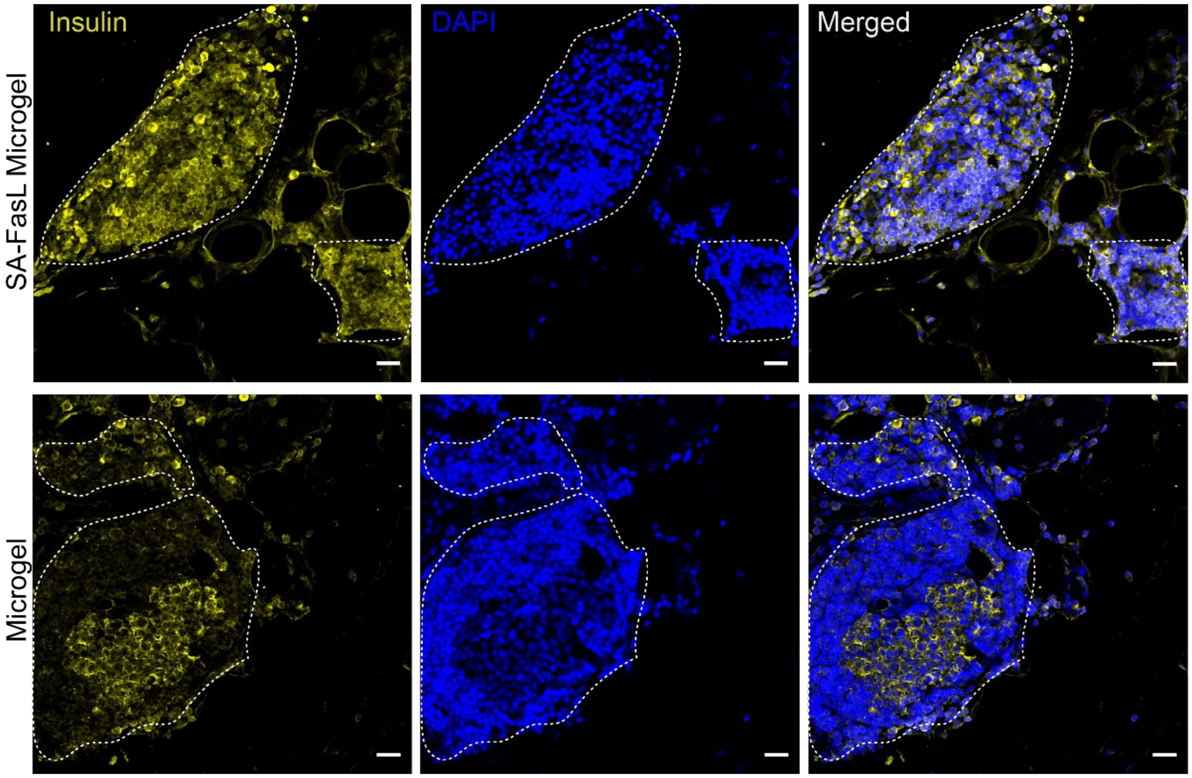

Fig. S7. Immunostaining analysis of grafts demonstrating insulin$^+$ structures in recipients of SA-FasL presenting microgels. Staining for insulin (yellow) and DAPI (blue) showing preserved insulin$^+$ structures (dashed lines) at the graft site (representative section from M518 at 177-day graft removal), whereas control Microgel section shows loss of insulin expression at the periphery of structure (representative section from M8918 at 21-day endpoint). Scale bar 20 µm.

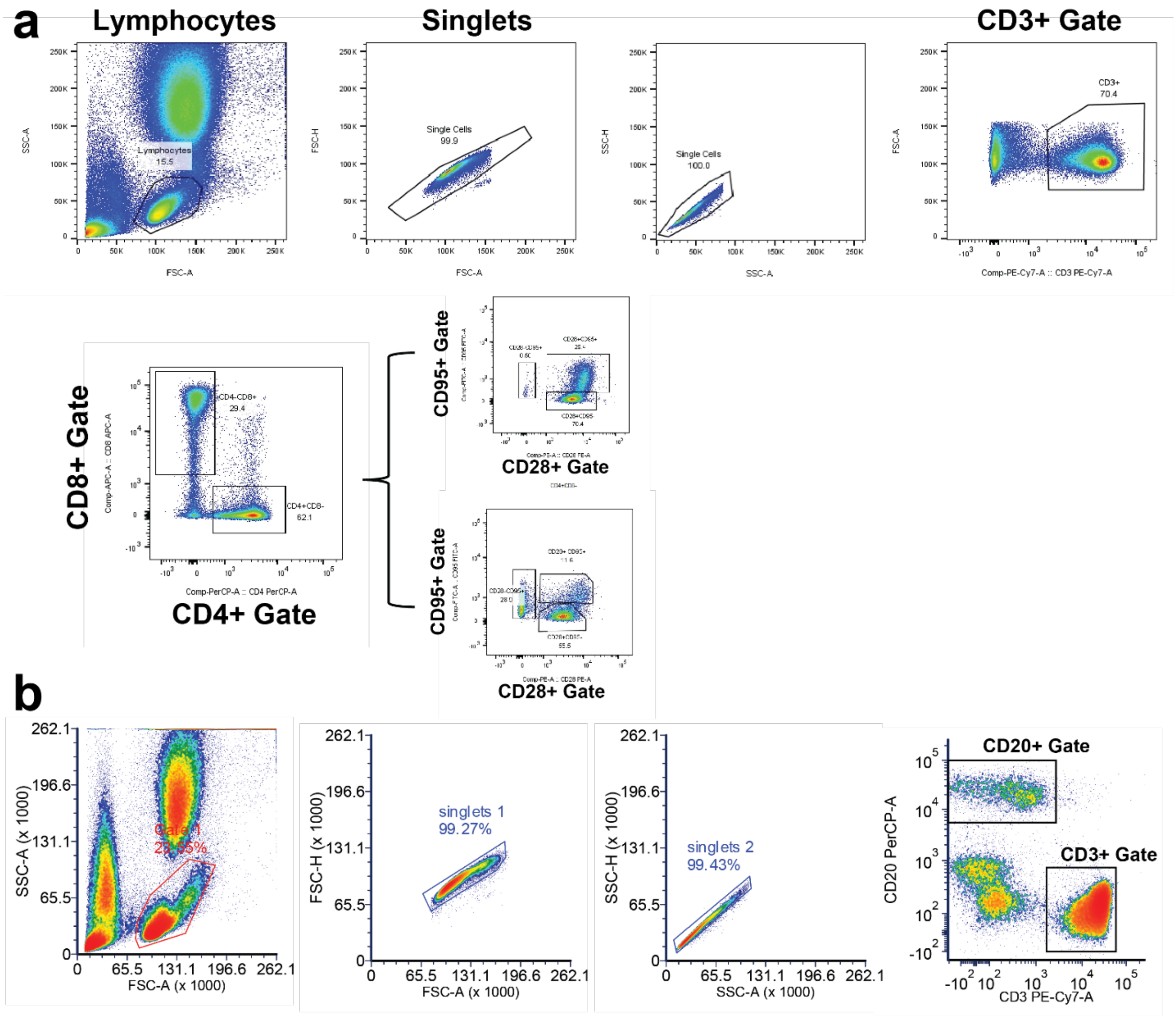

Fig. S8. Flow cytometry gating for peripheral blood lymphocyte profiling. a, Gating strategy for CD3$^+$, CD4$^+$, CD8$^+$, as well as naïve (Tn), effector memory (EM), and central memory (CM) CD4$^+$ and CD8$^+$ cells. **b**, Gating strategy for CD20+ cells.

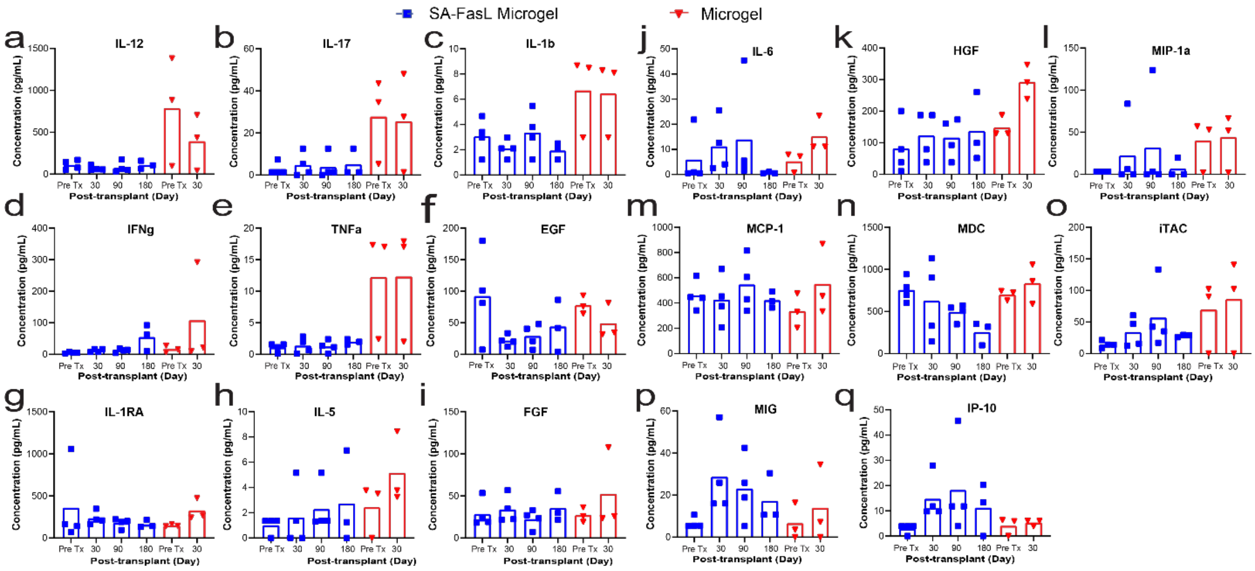

Fig. S9. Longitudinal tracking of serum cytokine and chemokine levels in animals receiving SA-FasL-microgels and control microgels. Serum chemokine and cytokine concentrations (mean, individual points) for subjects in receiving SA-FasL Microgels (blue, n=3-4) or control Microgels (red, n=3). At the preselected posttransplant time points, no differences (P>0.05) to pre-transplantation concentrations were detected using repeated measures ANOVA.

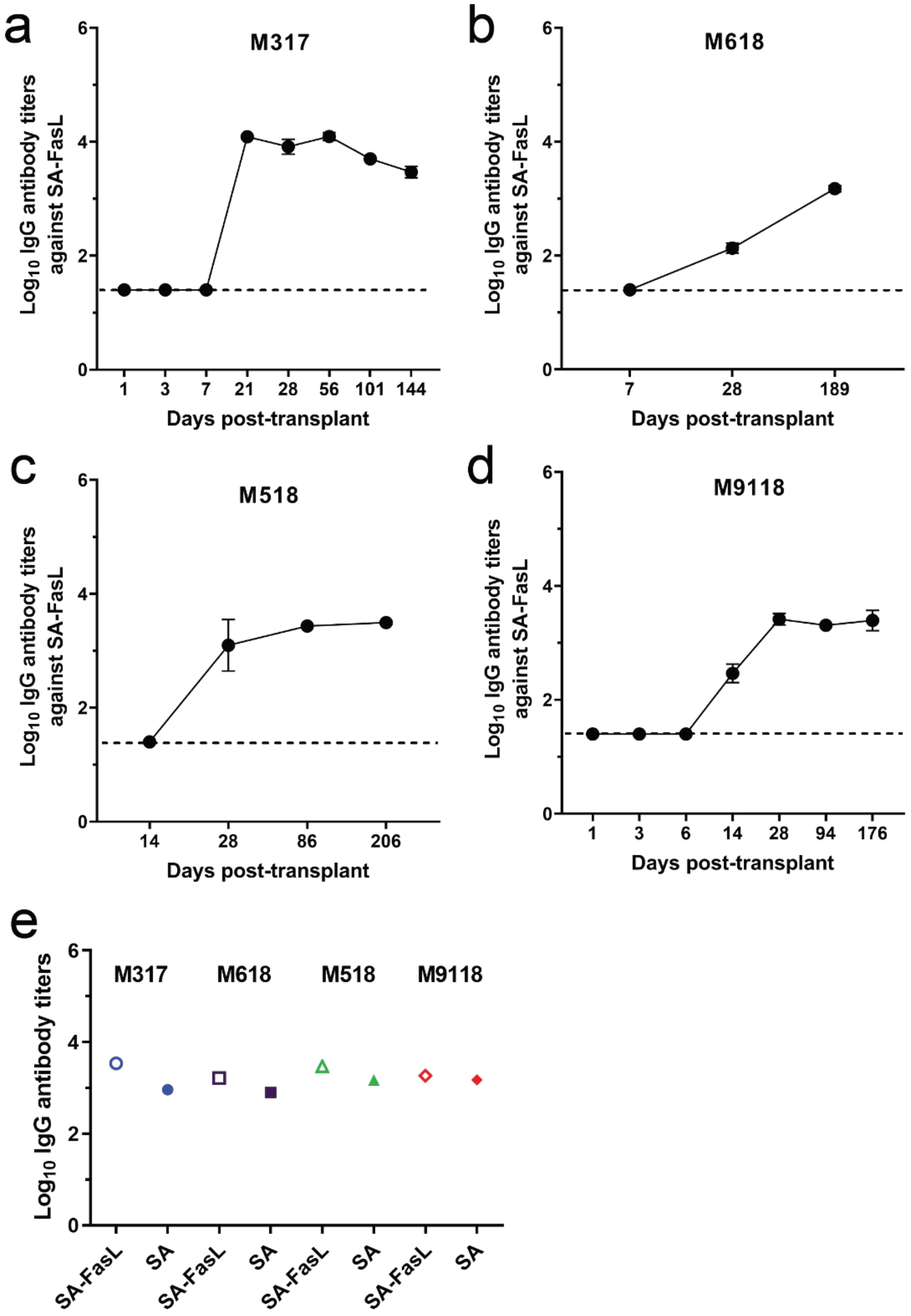

Fig. S10. IgG titers against SA-FasL in NHPs receiving SA-FasL-microgels. a-d, Serum samples obtained at different timepoints from subjects receiving SA-FasL Microgels were tested for antibodies for SA-FasL by ELISA. The maximum dilution of serum samples that gave a positive result in the ELISA was selected as the ELISA titer (dashed lines). **e**, IgG titers against the SA component vs. the whole SA-FasL molecule in endpoint serum samples.

a Summary of NHP Donors and Islet Assessment

Cyno ID	Weight (kg)	Pancreas Weight (g)	IEQ	Packed Cell Vol. (mL)	Islet Purity (%)	Recipient ID
M717	8.4	8.8	105,000	0.4	80	SA-FasL-Microgel 1
M418	7.1	8.1	93,500	0.5	50	SA-FasL-Microgel 2
M118	8.5	8.7	110,200	0.3	80	SA-FasL-Microgel 3
M6518	6.2	6.4	80,300	0.4	65	SA-FasL-Microgel 4
M1017	7.9	7.7	115,200	0.3	85	Microgel 1
M120	8.6	8.4	96,000	0.4	65	Microgel 2
M220	8.5	8.7	94,700	0.5	50	Microgel 3

b Summary of NHP recipient characteristics

Animal ID	Cyno ID	Weight (kg)	Transplant Dosage (IEQ/kg)	Baseline DSA (MFI)	
				Class I	Class II
SA-FasL-Microgel 1	M317	6.5	16,200	2326	2442
SA-FasL-Microgel 2	M618	6.3	14,800	3628	4526
SA-FasL-Microgel 3	M518	6.2	17,800	2027	2590
SA-FasL-Microgel 4	M9118	4.3	18,700	2027	2590
Microgel 1	M517	5.9	19,500	727	2892
Microgel 2	M8118	4.8	20,000	162	975
Microgel 3	M8918	4.9	19,300	177	1953

Table S1. Summary of donor and recipient subjects. **a**, Summary of NHP donors and islets, listing animal study ID number, weight of animal and pancreas prior to isolation. Islet yield and quality for each donor is provided as well as the intended recipient. **b**, Summary of recipient characteristics, listing animal ID numbers, weight and transplant dosage, immunosuppression used, and their class I and II DSA status.

a

SA-FasL Microgel	Recipient 1		Donor 1		Recipient 2		Donor 2		Recipient 3		Donor 3		Recipient 4		Donor 4	
MHC-A	M3	M3	M3	M4	M1A	M6A	M6A	M3A	M1A	M6A	M5A	M4A	M4A	M2A	M3A	M1A
MHC-B	M3	M3	M4	M6	M1B	M6B	M1B	M3B	M1B	M6B	M5B	M4B	M4B	M2B	M3B	M1B
MHC-DRB	M3	M3	M4	M6	M1DR	M6DR	M1DR	M3DR	M1DR	M6DR	M5DR	M3DR	M4DR	M1DR	M3DR	M1DR
MHC-DQA/B	M3	M3	M4	M6	M1DQ	M6DQ	M1DQ	M3DQ	M1DQ	M6DQ	M5DQ	M3DQ	M4DQ	M1DQ	M3DQ	M1DQ
MHC-DPA/B	M3	M3	M4	M4	M1DP	M6DP	M1DP	M1DP	M1DP	M6DP	M5DP	M3DP	M4DP	M1DP	M3DP	M1DP

Microgel	Recipient 1		Donor 1		Recipient 2		Donor D2		Recipient 3		Donor 3	
MHC-A	M1	M6	M2	M4	M1A	M5A	M4A	M5A	M1A	M2A	M1A	M3A
MHC-B	M1	M6	M2	M4	M1B	M5B	M4B	M5B	M1B	M2B	M1B	M3B
MHC-DRB	M1	M6	M2	M4	M1DR	M5DR	M4DR	M5DR	M1DR	M2DR	M1DR	M3DR
MHC-DQA/B	M1	M6	M2	M4	M1DQ	M5DQ	M4DQ	M5DQ	M1DQ	M2DQ	M1DQ	M3DQ
MHC-DPA/B	M1	M6	M2	M4	M1DP	M5DP	M4DP	M5DP	M1DP	M2DP	M1DP	M3DP

b

Summary of MHC Disparity					
**Animal ID**	**Class I A**	**Class I B**	**Class II-DRB**	**Class II-DQA/B**	**Class II-DPA/B**
SA-FasL Microgel 1	Haplo mm	Full mm	Full mm	Full mm	Full mm
SA-FasL Microgel 2	Haplo mm	Haplo mm	Haplo mm	Haplo mm	Haplo mm
SA-FasL Microgel 3	Full mm	Full mm	Full mm	Full mm	Full mm
SA-FasL Microgel 4	Full mm	Full mm	Haplo mm	Haplo mm	Haplo mm
Microgel 1	Full mm	Full mm	Full mm	Full mm	Full mm
Microgel 2	Haplo mm	Haplo mm	Haplo mm	Haplo mm	Haplo mm
Microgel 3	Haplo mm	Haplo mm	Haplo mm	Haplo mm	Haplo mm

Table S2. MHC typing of donor and recipient subjects. a, MHC haplotypes. **b**, Summary of MHC disparity. Full mm = full mismatch, Haplo mm = haplotype mismatch.
